# American Medical Association

**Published:** 1854-05

**Authors:** 


### EDITORIAL DEPARTMENT.

---

### AMERICAN MEDICAL ASSOCIATION.

It is already known to our readers that this body convened on the 2d inst. at St. Louis, but they may not be apprised of its proceedings. We will therefore endeavor to give a brief abstract from the reports, condensing as much as possible for the sake of brevity.

The first day was spent in preliminaries,—in effecting an organization, and appointing committees. The delegates from each state selected and detailed one of their number to form a general committee for the nomination of officers, and for the transaction of other business which might be referred to them by the Association. Our venerable friend Dr. Siviter of Salem, was first selected from this State, and as he returned home before adjournment, another was appointed in his place. The committee thus constituted, reported the names of gentlemen suitable for officers of the Association. Dr. Pope of St. Louis was chosen President, upon which report there was a motion of acceptance and adoption, which was carried.

When Dr. Pope took the chair, he returned his thanks to the members of the Association for their partiality in his selection. We will here say that he presided over the deliberations of that body with ability and efficiency. We had not met Dr. Pope before, but from what we heard and saw of the man on this occasion, we were led to form a very favorable opinion of him and also of the profession generally of that city.

The President announced that the reading and consideration of the annual reports of committees would now be in order. The following chairman of committees submitted abstracts of their reports of the diseases and epidemics committed to their investigation, as appearing in their several sections and districts.

Dr. F. Condie, of Philadelphia, on the Causes of Tubercular Disease, was not prepared to report, and requested further time.

Dr. Geo. B. Wood, of Philadelphia, on Diseases of Parasytic origin not being present, had sent a verbal request to be discontinued. His request was accordingly granted.

Dr. John A. Atlee, of Lancaster, Pa., on Epidemics of New Jersey, Pennsylvania, Delaware and Maryland, not being prepared to make a full report, requested to be continued on the same committee.

Dr. D. J. Cain, of Charleston, S. C., on Epidemics of South Carolina, Florida, Georgia and Alabama, read an abstract of his report. It was referred to the Committee on Publication.

Dr. W. L. Sutton, of Georgetown, Ky., on Epidemics of Tennessee and Kentucky. He had made a partial report, but of such meagre materials that he requested to be continued. His report was referred to the Committe of Publication when ready.

Dr George Mendenhall, of Cincinnati, Ohio, on the Epidemics of Ohio, Indiana and Michigan. He presented a report for the years 1852 and 1853, of which he read a brief abstract. The report was referred to the Committee on Publication, with the request to have it published in the proceedings of the present year.

Drs Palmer and Atlee each spoke at some length of the great and lasting good that might be accomplished if all the members of the Association would duly record their individual experience of epidemics, and report all such cases to the Chairmen of the Committees. Dr Atlee had been two years Chairman of such a committee and during that time had only received two such reports,—one from New York, and a partial one from Pennsylvania. He stated that he had used great exertions to get professional men to co-operate in this work, and appealed to the whole faculty to do everything in their power to promote this great object. He very ably impressed the necessity of co-operation by all the profession with the several committees having these reports in charge.

Dr R S Holmes, of St Louis, Mo., on Epidemic Erysipelas read an abstract of his report. It was referred to the Committee on Publication.

Dr E D Fenner, of New Orleans, on Epidemics of Louisiana, Mississippi, Texas and Arkansas. He read a comprehensive abstract of his report—dwelling principally on the ravages of the cholera and yellow fever, and the causes and the means of treatment.

Dr Fenner had not completed his report, and Dr McPheeters offered a resolution that Dr Fenner be requested to complete his report, and submit it to the Committee on Publication to be published. The resolution was adopted.

Dr Mussey, of Cincinnati, now made a motion to suspend the order of regular business, to allow Dr Linton, of St Louis, to express his views with regard to the Pathology of the Yellow Fever. A suspension of business was made for this purpose.

He expressed his views very clearly and at some length on this subject. He advocated the idea that vegetable decomposition was not necessary to the production of the autumnal diseases of this country. He considered yellow fever nothing more than an aggravated type of bilious fever, caused by the retention of hydro-carbonaceous substances in the blood. In other words, the agencies producing yellow fever were Northern blood subject to the heat of Southern latitudes.

A motion was made and carried, that Dr Linton be requested to draw up the substance of his remarks, to be presented to the Committee of Publication.

Dr Dan'l Brainard of Chicago, Illinois, on the Constitution and Local Treatment of Carcinoma. He requested further time to make a full report.

Dr N S Davis of Chicago, Illinois, on the Influence of Local Circumstances on the Origin and Prevalence of Typhoid Feuer. The report, of which he read a brief abstract, was referred to the Committee on Publication.

Dr. Donaldson of Baltimore, on the Present and Prospective value of the Microscope in Disease. Dr Donaldson, in a communication, stated that his report was complete, but he not being present, it was, without reading, referred to the Committee on Publication.

The report of the Committee on Medical Education was received, but owing to its length, its reading was passed over. It was referred to the Committee of Publication.

Dr Pope, Chairman of the Committee on Prize Essays and Volunteer Communications, now made the following report:

Mr President: The Committee on Prize Essays and Volunteer Communications, respectfully report that the Essays submitted to their consideration were nine in number, of which one was presented as a volunteer communication. The committee have carefully examined the whole of these Essays and bestowed upon them the attention which a sense of the importance of the duty assigned them imposed. They feel free to say that some of these Essays possess undoubted merit, both in matter and style, and they admit in them evidence of high scientific attainment as well as a familiarity with the graces of composition. But whilst cheerfully according these claims to their authors, the committee have preferred to be governed in the choice, by considerations of originality and practical import, rather than of mere theoretic speculation, however finely portrayed. The committee have consequently concluded to award but a single prize. The Essay selected is entitled "An Essay on a new method of treating Ununited Fractures and certain Deformities of the Osseous System." It bears a motto in French, which, being liberally rendered in modern English, reads, "and notwithstanding all the pains I have heretofore taken, I have reason to praise God, in that it hath pleased Him to call me to that branch of Medical practice commonly called Surgery, which can neither be bought by gold nor by silver, but by industry alone and long experience.''

If it please the Association I will now break the seal of the packet superscribed by the same motto, and declare the name of the successful competitor.

Dr Pope then broke the seal and announced the name of Professor Brainard of Chicago.

Dr McPheeters moved that Prof Daniel Brainard take the stand and give the Association an abstract of his new mode of treating fractures, &c., which motion was carried, and Prof Brainard accordingly came forward, and in an able manner gave the requisite information.

Dr Hooker, of Connecticut, Treasurer, now introduced the subject of annual assessments, and called the attention of the Association to the fact that he was ready to receive the dues of members.

Dr Elbert offered certain resolutions to the effect that a committee be appointed to recommend to the next annual meeting, for consideration, any alteration they might deem necessary in the Constitution, By-Laws, &c., and also that the place of holding future annual meetings of the Association be determined by ballot, without the intervention of the Nominating Committee. The resolutions, after much discussion, were lost.

Dr Guthrie offered the following resolutions, which were unanimously carried:

*Resolaed*, That in the Secretary of the Treasury's recommendation to Congress to abolish or materially modify the duty on such crude drugs not producable in this country, as are used in the laboratories of the country in the manufacture of chemicals, we recognise a wise provision for the further protection of the profession and the community at large, from impure and sophisticated medicines.

*Resolved*, That a copy of this resolution be signed by the proper officers of this Association, and transmit the same to the Secretary of the Treasury and to the Committee on Ways and Means.

Dr J B Johnson now stated to the meeting that he had a letter from Dr Stephen Williams of Illinois, enclosing a preamble and resolution, which he desired to read to the meeting. He then read the letter, of which the following is a copy:

*Amerscan Medical Biography, for the American Medical Association, assembled at St. Louis.*

Mr President: At the last meeting of the Association at New York, I presented the following preamble and resolution through my friend Dr Stewart of New York, which, with some amendments, were laid on the table:

"As we are constantly called upon to deplore the ravages of death among the illustrious and worthy members of our profession throughout the United States,

*Resolved*, That a standing committee be appointed by this Association to procure memorials of the eminent and worthy dead among the distinguished physicians of our country, and present them to this Association for publication in the transactions.

I now beg leave to call up the resolution through my friend Dr J B Johnson, of St Louis. The medical biography of our country is intimately related to the history of it, as the lives of eminent men are identified with the history of the times in which they lived. In the United States there have been, and are to be found, medical men whose lives and actions are ornaments to human nature, and whose brilliant career in the cause of humanity and science reflect honor and dignity upon our country. We differ nothing in this respect in comparison with the learned and eminent physicians in Europe.

The profession of medicine contains more learned and distinguished men than any other profession or calling, and some memorial of their lives and actions should be presented to the world in a more durable form than the periodicals of the day, and particularly of the newspaper press. A more permanent and proper place for the publication of such memorials, would be in the Transactions of the American Medical Association, and without disparagement to any other articles which have heretofore been published in these Transactions—which memorials would be read by the members of the profession with great interest and improvement. Short biographies need take up but little room in the publication, and this objection to the proposed movement may be obviated. We are constantly noticing that death spares no rank or condition of men. Those who contend most skillfully against his insatiate ravages, themselves fall victims to his all conquering sword. Within a very short space of time, we have been called to lament the death of Dr Nathan Chapman, the former President of this Association, of Dr Samuel G Morton, Wm E Holmes, Isaac Parish, G S Pattison, J Kearney Rodgers, Daniel Drake, the great medical pioneer of the West, Samuel McClellan, Amos Switchell, Abiel Pierson, G C Shattuck, Archibald Welch, and very many others which time will not permit me to enumerate.

This is not the place to speak their eulogies. Some permanent notice of them and of many others who have recently died, should be published in the Transactions of this Association, where the useful improvements and discoveries of the living should be recorded, and the memories of the worthy dead should be preserved. I hope the resolution will prevail.

[Signed,] STEPHEN W WILLIAMS.

Lavina, Winnebago county, Illinois, late of Deerfield, Massachusetts.

The Preamble and Resolutions referred to in the foregoing, were then on motion adopted. The Chair then announced that he would appoint the Committee contemplated by the resolution, hereafter.

Dr McIlvaine offered the following rosolution:

*Resolved*, That in the opinion of this Association, the practice of Professors reading lectures to their classes, no matter with how much care selected from the musty records of antiquity, is a miserable apology for teaching, is prima facie evidence of their inaptness to instruct, and is inimical to medical progress.

It was, on motion, laid on the table.

Dr French submitted the following resolution, which was carried :

*Resolved,* That a committe be appointed to enquire what State or other Society, represented in this Association, are in fellowship with irregular practitioners.

Dr Blachford of Troy, read a letter from Dr A D Spore, stating that he (Dr Spore) had been for some time investigating the subject of Hydrophobia, to ascertion what influence the weather had upon the disease, and in the letter he requested that communications on the subject might be sent to him by members of the Faculty who had opportunities of making observations. Dr Spore not being a Delegate, it was moved that Dr. Blachford be appointed Chairman of a Committee for the investigation of this subject.

The following resolution was offered by Dr McDowell:

*Resolved,* That a committee be appointed to investigate the improvements of the instruments for Lithotomy by Nathan R Smith, Paul F Eve and McDowell.

The resolution was laid on the table.

The following was offered by Dr S M Smith, of Columbus, Ohio, which was carried :

*Resolved,* That a Standing Commttee of —— be appointed by the Association on the subject of Insanity as it prevails in this country, including its causation as hereditary transmission, educational influences, physical and moral, social and political institutions, &c. Its forms and complications, curability and means of prevention.

### AFTERNOON SESSION.

The Association met as per adjournment.

Dr Samuel P White, of the University of Buffalo, submitted the following resolution, which was carried :

*Resolved,* That the thanks of this Association be presented to Dr J Knight, late President, for the very dignified, courteous and efficient manner in which he presided over its deliberations, and that he be respectfully requested to furnish the usual address for publication.

The committee appointed by the American Medical Association to devise or consider some comprehensive plan for the more general, systematic and thorough investigation of subjects connected with medical science, made a report, to which was appended the following resolution :

*Resolved,* That the American Medical Association hereby recommends all Medical Societies to establish, in accordance with the plan detailed in the foregoing report, special committees for the selection, investigation, callaboration and publication of all subjects of interest connected with medical science.

The resolution carried, and the report and resolution was referred to the Committee of Publication.

Dr Atlee communicated to the meeting that he had received a letter from Dr Parish, Chairman of the Committee on Epidemics of New Jersey, stating that his report was yet unfinished, but would soon be ready for publication.

On motion, it was directed to be handed over to the Committee of Publication when finished.

Mr Davis presented some specimens of milk to the Association, which he explained would, if used, prevent many of the diseases to which children are subject, arising from using putrid milk. He respectfully submitted the specimens to the consideration of the Association.

### THIRD DAY—THURSDAY.

The Association convened at nine o'clock, A M. Dr Pope, President, in the chair.

On motion, the regular order of business was suspended for the purpose of filling the vacancy in the Nominating Committee from Iowa.

On motion, Dr M'Gugin was chosen to fill the vacancy.

The minutes of Wednesday's proceedings were read, and after a few unimportant amendments, were adopted.

Dr Atlee offered the following resolutioa, which carried :

*Resolved,* That it shall be the duty of the Publication Committee to append to each volume of the transactions hereafter published, a copy of the Constitution of the Association.

The following resolution, offered by Dr Gross, was also carried, and Dr Gross was appointed by the Chair the committee designated :

*Resolved,* That a committee of one be appointed by the Chair to inquire into the causes which obstruct the formation and establishment of our National Medical Literature, and to report the subject at the next annual meeting of this Association, or as soon thereafter as practicable.

Dr Paul F Eve, of Nashville, Tenn., submitted a resolution, which, after amendment, as follows, was carried :

*Resolved,* That a committee of three be appointed by the Chair, to report at the next meeting of the Association, the best means for preventing the introduction of disease by emigrants into our country.

The Chair appointed Drs Dickson, Griscom and E D Fenner, a committee.

Dr Linton, of St Louis, offered the following, which was also referred to the above named committee :

*Resolved,* That in the opinion of this Association, quarantine establishments afford no protection to States and cities against the invasion of epidemics such as cholera and yellow fever.

### REPORT OF THE COMMITTEE OF NOMINATIONS.

The Committee on Nominations, in fulfilling the duty imposed upon them, recommend the continuance of several of the special committees previously created, and the appointment of some new ones.—They therefore, submit the following list of Chairmen of special committees, with the subjects to them committed :

Dr Worthington Hooker, of New Haven, Connecticut, on epidemics of New England and New York.

Dr John L Atlee, of Lancaster, Pa., on epidemics of New Jersey, Pennsylvania, Delaware and Maryland.

Dr D J Cain, of Charleston, S C., on epidemics of South Carolina, Florida, Georgia and Alabama.

Dr W L Sutton, of Georgetown, Ky., on epidemics of Tennessee and Kentucky.

Dr Thos Reyburn, of St Louis, Mo., on epidemics of Missouri, Illinois, Iowa and Wisconsin.

Dr Geo Mendenhall, of Cincinnati, Ohio, on epidemics of Ohio, Indiana and Michigan.

Dr E D Fenner, of New Orleans, La., on epidemics of Mississippi, Louisiana, Arkansas and Texas.

Dr James Jones, of New Orleans, La., on the mutual relations of yellow and bilious remittent fever.

Dr D V Condie, of Philadelphia, Pa., on the causes of Tubercular Disease.

Dr Jos Leidy, of Philadelphia, Pennsylvania, on diseases of the Parastic Origin.

Dr A P Merrill, of Memphis, Tenn., on the Physiological Peculiarities and Diseases of Negroes.

Dr Jos N McDowell, of St Louis, Mo., on Statistics of the operation for the Removal of Stone in the Bladder.

Dr F P Porcher, of Charleston, S C., on the Toxicological and Medicinal Properties of our Cryptogamic Plants.

Dr Daniel Brainard, of Chicago, Illinois, on the Constitutional and Local Treatment of Carcinoma.

Dr Geo Engleman, of St Louis, Mo., on the Influence of Geological Formations on the Character of Disease.

Dr Henry Taylor, of Mount Clemens, Mich., on Dysentery.

Dr Horace Green, of New York, on the use and effect of applications of Nitrate of Silver to the Throat in Local or General Disease.

Dr P C Gooch, of Richmond, Va., on the administration of Anæsthetic Agents, during Parturition.

Dr Charles Hooker, of New Haven, Connecticut, on the Diet of the Sick.

Dr E R Dabney, of Clarksville, Tenn., on certain forms of Eruptive Fevers, prevalent in Middle Tennessee.

Dr Sanford B Hunt, of New York, on the Hygrometrical State of the Atmosphere in various localities, and its influence on health.

Dr. Frank H Hamilton, of Buflalo, New York, on the frequency of deformities in fractures.

Dr M M Pallen, of St Louis, Mo., on diseases of the Prostrate Gland.

Dr H A Johnson, of Chicago, Illinois, on the Excretions as an index to the Organic Changes going on in the system.

Dr Leroy H Anderson, of Sumpterville, Ala., on Typhoid Fever and its complications as it prevails in Alabama.

Dr W H Byford, of Evansville, Ia., on the Pathology and Treatment of Scrofula.

Dr N S Davis, of Chicago, Ills., on the Nutritive Qualities of Milk, and the influence produced thereon by pregnancy and menstruation in the human female, and by pregnancy in the cow, and also on the question whether there is not some mode by which the nutritive constituents of milk can be preserved in their purity and sweetness, and furnished to the inhabitants of cities in such quantities as to supercede the present defective and often unwholesome method of supply.

Dr E B Haskens of Clarksville, Tenn., on the Microscopical Investigations of Malignant Tumors.

Dr Geo K Grant, of Memphis, Tenn., on the Sulphate of Quinia as a Remedial Agent in the treatment of Fevers.

Dr R R McIlvain of Cincinnati, Ohio, on the Study of Pathology at the Bedside.

Dr E S Cooper, of Peru, Ills., on Orthopædic Surgery.

Dr Andrew F Jeter, of Palmyra, Mo., on the Modus Operandi of the Envenomed secretions of healthy Animals.

Dr Sam'l M Smith, of Columbus, Ohio, on Insanity.

Dr Rene La Roche, of Philadelphia, Penn., on the Jaundice of Yellow Fever in its Diagnostical and Prognostical Relations.

Dr Charles Chandler, of Rocheport, Mo., on Malignant Periodic Fevers.

Dr S B Chase of Portland, Maine, on Typhoid Fever in Maine.

Committee on Plans of Organization for State and County Societies—A B Palmer, M D., Michigan ; R R McIlvain, M D., Ohio ; D L M'Gugin, M D., Iowa ; E R Peaslee, M D., New Hampshire ; Thos Lipscomb, M D., Tennessee.

Committee on Medical Literature—Robert J Breckenridge, M D., Kentucky, Chairman ; A A Gould, M D., Mass. ; D L M'Gugin, M D., Iowa ; J B Flint, M D., Ky. ; O M Langdon, M D., Ohio.

Committee on Medical Education—Wm H Anderson, M D., Alabama ; A Lopez, M D., do. ; Andrew Murray, M D., Michigan ; A Ramsay, M D., Tennessee ; R D Ross, M D.

Committee on Prize Essays—Rene La Roche, M D., Pennsylvania ; Isaac Hays, M D., do. ; Alfred Stile, M D., do. ; J B Biddle, M D., do. ; Geo W Norris, M D., do. ; Joseph Carson, M D., do. ; Joseph Leidey, M D., do.

Committee of Arrangements—Isaac Hays, M D., Pensylvania ; G Emerson, M D., do. ; Wilson Jewell, M D., do. ; Alfred Stille, M D., do. ; Francis West, M D., do. ; Wm V Keating, M D., do.

Committee on Publication—Pliny Earle, M D., New York ; D Francis Condie, M D., Pennsylvania ; E S Lemoine, M D., Missouri ; A March, M D., New York ; E H Davis, M D., do. ; C R Gillman, M D., do.

Dr Atlee offered the following resolution, which was carried:

*Resolved,* That this Association earnestly recommeud to their medical brethren in those States in which Societies do not exist, the immediate organization of State and County Medical Societies.

Dr Ramsay offered a resolution, which, on motion of Dr Coons, was laid on the table.

Dr Breckenridge offered a resolution to the following effect:

*Resolved,* That the papers and documents of the Association shall hereafter be the exclusive property of the Association, which, as amended by Dr Smith, was carried.

From this State :—Drs Elbert, Siviter, Rauch, Van Patten and Arnold from the State Medical Society—Drs M'Gugin and Ford from the Medical College—Dr Ely from the Society North, and Dr Hughes from the Hospital, Dr Sanford a member and Dr Walker by invitation.

A resolution was offered by Dr. Gross, the design of which was to discourage festivities and public entertainments, was adopted. The practice has imperceptibly crept into these convocations and has become by custom, rather a part of the proceedings. It had its origin in Charleston, S. C., a city remarkable for the hospitality of her citizens. Other cities followed suit and each in turn was disposed to excel the former in the richness, splendor and magnificence of its entertainments. The recent display on the part of St. Louis, cannot be very well excelled, and we hope now that they will be discontinued *indefinitely*. This was the ardent wish of every member with whom we conversed. All spoke in the highest terms of the sumptuous repasts of St. Louis, public and private, and of the noble and generous disposition manifested by all, both in the profession and out of it, to make the stay of the delegates agreeable and pleasant.

There are several cogent objections to these banquets. One is that the time of the delegates is too much engrossed by them to leave any share for maturing and deliberatiug upon the questions to be brought before the Association, and those actually under consideration. Questions of grave import should undergo that scrutiny which their importance demands, and as the Association had its origin in motives of mutual and reciprocal benefit, in the hope that the cause of medical science might be advanced, the best interests of the profession promoted, that each member would come up yearly with the rich fruits of his past experience and observation and invite his friends to a participation in the choice products of his labors. This should be the feast, the *banquet* at which the mind is to be regaled, and Science the Goddess, in whose honor these libations should be poured. If we are right in our estimate of the motive and design of the organization of the Association, the time should be consumed in the discussion and settlement of doubtful points or in the establishment of proximal views. We can then be advancing *toward* truth at least, if we do not positively attain to it.

Above all men in society, the members of the profession have a moral duty to discharge in precept and by example. Our duty is not confined to the cure of ills nor simply in the alleviation of suffering. We are required to raise our warning voice and instruct our fellow men how they may prevent disease, or if they cannot prevent it, to rob it of some of its exaggerated inflictions. Hygienic rules should be given and dietetic laws enforced. Our precepts must possess the virtue of sincerity, if they avail aught, and our practice is the best evidence of the purity of our purpose. But how sadly did our practice falsify all claims to sincerity at the recent and several of the former meetings of the Association. The forest of bottle necks, the poping of champaigne bottle corks, with other demonstrations of which "this deponent saith not" were circumstances, in no way in accordance with the prevailing sentiment in which medical men should be first and prominent, nor flattering to the dignity of the profession. *Verbum sapientiæ sufficit*, and we hope that in the future our conduct will be more in accordance with the position that medical men occupy in community, and the professions we make on all occasions of sobriety and abstinance.

